# University of Pennsylvania

**Published:** 1899-12

**Authors:** Harry E. Bender

**Affiliations:** Secretary


### VETERINARY MEDICAL SOCIETY, UNIVERSITY OF PENNSYLVANIA.

The meeting was called to order Friday evening, October 20th, at 8 o'clock, by the Secretary, Mr. Nolan. Mr. Bender was appointed Secretary *pro tem.*

Mr. Young was made critic. It was moved, seconded, and carried to dispense with the regular business and to proceed to the proposal and election of new members. The names of Messrs. Weitzel, '02, Burrows, '02, Bigelow, '02, McClintock, '02, Nicholas, '02, Zollinger, '02, Rothermel, '02, Cole, '02, Glass, '02, Paget, '02, Feigenbaum, '02, McCloskey, '02, were proposed and duly elected to membership.

The next in the order of business was the nomination and election of new officers. Messrs. Horner, Cornman, and Nolan were nominated for President. Messrs. Young and Hughes for Vice-President. Messrs. Walters and Bender for Secretary. Messrs. Davison, Mayer, Stehle, Tallman, and White for Treasurer. Messrs. Powell, Mayer, Carlisle, Basler, Shore, Watson, Norton, Zollinger, Feigenbaum, and McCloskey for the Executive Committee.

Mr. Hayman was nominated for Librarian.

The election resulted as follows: President, Mr. Nolan, '00; Vice-President, Mr. Young, '00; Secretary, Mr. Bender, '01; Treasurer, Mr. Davison, '00; Librarian, Mr. Hayman, '01; Executive Committee, Messrs. Powell, '00, Carlisle, '01, Shore, '01, and McCloskey, '02.

After the result of the election was announced the newly elected officers took their seats.

The critic had no report to make. Adjourned at 9.30 P.M.

The meeting was called to order November 3, 1899, at 8 o'clock P.M. Mr. Horner was appointed critic.

It was moved, seconded, and carried that the names of such persons which are on our roll at present and who are no longer in attendance at the school be stricken off our roll.

Dr. Adams then addressed the meeting on "The Method of Fitting Bits in Horses' Mouths." Dr. Adams made his lecture very interesting by the way in which he described the various forms of bits and showing the position they should occupy in the mouth. A vote of thanks was extended to Dr. Adams.

The critic had no report to make. Adjourned at 9.15 P.M.

The meeting was called to order November 17, 1899, at 8 o'clock P.M. Mr. Mayer was appointed critic.

The Chair appointed the following Room Committee: Messrs. Hughes, '00, Bassler, '01, and Paget, '02.

It was moved, seconded, and carried that the Librarian be authorized to have 100 copies of the Constitution and By-laws of the Society printed.

Mr. Cornman then read a paper entitled "Ectopia Cordis," which was very interesting. He also had on hand a specimen which showed it very nicely. A vote of thanks was extended to Mr. Cornman.

The critic had no report to make. Adjourned at 9.15 P.M.

HARRY E. BENDER,
Secretary.

